# Editorial: Organogenesis: From Development to Disease

**DOI:** 10.3389/fcell.2017.00085

**Published:** 2017-09-20

**Authors:** Sunder Sims-Lucas, Misty Good, Seppo J. Vainio

**Affiliations:** ^1^Pediatrics, Children's Hospital of Pittsburgh Pittsburgh, PA, United States; ^2^Pediatrics, Washington University in St. Louis St. Louis, MO, United States; ^3^Laboratory of Developmental Biology, Biocenter Oulu, InfoTech Oulu, Faculty of Biochemistry and Molecular Medicine, Oulu University Oulu, Finland

**Keywords:** animal, inflammation, kidney development, bowel disease, diabetes mellitus, type 1, bladder innervation, bone mineralization

The formation of the developing fetus is under strict molecular and cellular control. These developmental processes have to coordinate seamlessly to produce a viable and healthy fetus. When these processes go astray this can lead to dramatic defects to the embryonic tissue and even to the death of the embryo. These miscues can be due to genetic or environmental influences. Even slight malformations of the organs can cause developmental reprogramming and increase the likelihood of adult onset diseases. The use of experimental animals with genetic mutations that mimic many human conditions has lead to significant scientific advancement of these complex and multifaceted diseases. Furthermore, the utilization of organ culture systems has made for profound insights into the formation of organs. Over recent years, the technology related to molecular profiling and imaging of developing organs has dramatically improved, leading to the identification of subtle genetic and phenotypic alterations. The major objectives of this topic was to explore the vast array of research that is focused on the formation of tissue and how this relates to the production of healthy tissue.

This editorial highlights the diverse nature of organ development and the exciting cross-disciplinary approaches that can be employed to answer questions related to the origin of developmental abnormalities, treatments, and post-natal susceptibility. This series of publications is made up of a combination of original science, reviews and mini-reviews.

The first original article describes the protective role of immunostimulated arginase expressing intestinal epithelial cells, which may be critical in the treatment of necrotiing enterocolitis (NEC). This is a highly important area of research as the incidence of NEC has not changed in the last several decades and the mortality rate can approach 50%. The treatment strategies to attenuate the exaggerated inflammation in this devastating disease are limited and the authors define an important mechanism that may be leveraged for therapeutic benefit (Talavera et al.).

The next original article focuses on establishing a mathematical model for bone mineralization (Komarova et al.). Mineralization is critical for the structural integrity of bone and without appropriate calcification the bones are fragile and prone to fractures and breaks. Up until now there has not been an effective characterization of bone mineralization. This manuscript has developed innovative algorithms to map the various stages of bone mineralization that may be applied to patients with genetic defects to aid in their personalized treatments.

There is a series of reviews on kidney formation including an article interrogating the multifaceted role of beta catenin in the kidney (Boivin et al.). Beta catenin signaling is one of the critical building blocks of kidney development and is an important signaling pathway involved in the pathogenesis of renal dysplasia. Together with the various Wnt ligands this signaling cascade is essential for almost every component of the developing kidney. The second kidney review focuses on the current approaches to generate functional kidney tissue (Hariharan et al.). The field has been moving toward the generation of personalized therapies for each individual patient. This is largely driven by the generation of kidney cells and organoids from a patient's own cells. This review discusses the current state of stem cell reprogramming and differentiation toward a kidney fate. Finally, the last kidney review investigates the utilization of circulating exosomes as diagnostic markers and therapeutic agents (Krause et al.). Kidney disease leading to end stage kidney failure is one of the fastest growing disease processes and is further driven by the rising numbers of diabetic and obese patients. The therapies for kidney disease are limited and often the disease presents very late at an irreversible stage. This review details the exciting field of exosomes and describes their role in development and disease. Further, it discusses their utilization as a potential biomarker and vehicle for therapeutics.

Following is a review on the formation of the bladder, with a particular focus on the innervation of the bladder (Keast et al.). The primary function of the bladder is to store and excrete urine, which is controlled by the sympathetic and parasympathetic nervous systems. This is driven by a complex and detailed innervation pattern controlling the bladder muscle and the various genitourinary openings. This review details the innervation and localization of the neuronal bundles throughout development utilizing various rodent models.

There are two review articles that focus on the bowel the first looking at the role of the cytokine interleukin 22 (IL-22) (Parks et al.). Data is emerging regarding the precise role of IL-22 in the intestine. This review summarizes the current data on the role of IL-22 as an inhibitor of inflammation and also as a key player in the gut barrier defense against various pathogens. The second review focuses on the mitochondria in inflammatory bowel disease (Novak and Mollen). Inflammatory bowel disease is a complex and multifactorial disorder with unknown etiology. Typically, there is destruction of the epithelial layers of the gastrointestinal tract, increased permeability through these damaged layers and an influx of inflammatory cells. These epithelial cells are highly metabolically active and the primary engine of this energy source is the mitochondria. This review delves into the mitochondrial damage that is observed in inflammatory bowel disease and discusses whether this dysfunction is the cause or a readout of the damage in these patients.

The final review looks at the role of β cell endoplasmic reticulum (ER) stress in the context of Type 1 diabetes (Marre et al.). Type 1 diabetes is a chronic autoimmunity disorder as the β cells are damaged and unable to produce insulin, the glucose levels continue to climb leading to further damage. ER stress is a common attribute to the β cell as a part of its normal physiological role of insulin generation. This review highlights the specific role of ER stress in triggering the autoimmune response and suggests that this may be a sufficient mechanism in driving this disease progression.

This series of original articles and reviews has stimulated significant interest and impact in the field of organogenesis. It is exciting how each field draws parallels that can be applicable across the biomedical sciences and various disease processes. We would like to thank the authors for their valuable contributions and the thought provoking manuscripts that they have produced.

## Author contributions

SS wrote the entire first draft of the manuscript and performed the editing before submission. MG and SV edited the first draft and provided intellectual input.

### Conflict of interest statement

The authors declare that the research was conducted in the absence of any commercial or financial relationships that could be construed as a potential conflict of interest.

